# Amyloid-β slows cilia movement along the ventricle, impairs fluid flow, and exacerbates its neurotoxicity in explant culture

**DOI:** 10.1038/s41598-023-40742-0

**Published:** 2023-08-21

**Authors:** Ryota Makibatake, Sora Oda, Yoshiki Yagi, Hitoshi Tatsumi

**Affiliations:** Department of Applied Bioscience, Kanazawa Institute of Technology, Hakusan-shi, Ishikawa 924-0838 Japan

**Keywords:** Cell death in the nervous system, Cellular neuroscience, Circadian rhythms and sleep, Diseases of the nervous system

## Abstract

Alzheimer’s disease (AD) is characterized by extensive and selective death of neurons and deterioration of synapses and circuits in the brain. The Aβ1–42 concentration is higher in an AD brain than in cognitively normal elderly individuals, and Aβ1–42 exhibits neurotoxicity. Brain-derived Aβ is transported into the cerebrospinal fluid (CSF), and CSF flow is driven in part by the beating of cilia and CSF secretion into ventricles. Ventricles are lined with ependyma whose apical surface is covered with motile cilia. Herein, we constructed an experimental system to measure the movement of ependymal cilia and examined the effects of Aβ1–42 to the beating of cilia and neurons. The circadian rhythm of the beating frequency of ependymal cilia was detected using brain wall explant-cultures containing ependymal cilia and neurons; the beating frequency was high at midday and low at midnight. Aβ1–42 decreased the peak frequency of ciliary beating at midday and slightly increased it at midnight. Aβ1–42 exhibited neurotoxicity to neurons on the non-ciliated side of the explant culture, while the neurotoxicity was less evident in neurons on the ciliated side. The neurotoxic effect of Aβ1–42 was diminished when 1 mPa of shear stress was generated using a flow chamber system that mimicked the flow by cilia. These results indicate that Aβ1–42 affects the circadian rhythm of ciliary beating, decreases the medium flow by the cilia-beating, and enhances the neurotoxic action of Aβ1–42 in the brain explant culture.

## Introduction

Alzheimer’s disease (AD) is neuropathologically characterized by the deposition of intracellular tau aggregates, extracellular amyloid beta (Aβ) deposits, and loss of synapses^1^. Aβ is derived by proteolytic processing of amyloid precursor protein (APP), resulting in a peptide predominantly 40 or 42 amino acids in length. Aβ1–42 is more fibrillogenic than Aβ1–40, and fibrillization of Aβ1–42 becomes amyloid deposits in the brain parenchyma^2^. The Aβ1–42 concentration is higher in AD brains than cognitively normal elderly individuals^3^, and in the cerebrospinal fluid (CSF) (or plasma)^4–6^. The clearance rates for Aβs are lower in subjects with AD compared with control subjects^7^.

There are several clearance pathways of Aβs in the mammalian brain: phagocytosis, endocytosis, macropinocytosis by different cells (e.g., microglia, astrocytes, and neurons), proteolytic degradation by enzymes (e.g., neprilysin), and efflux of Aβs to the peripheral circulation^3^. CSF and fluorescently-labeled amyloid-β are transported along the drainage pathways of the paraventricular veins^8,9^. The ependymal cells lining the ventricular space have motile cilia, and the defects in the motile cilia obstruct CSF flow^9^, and affect CSF production at the choroid plexus^10^. Ependymal cells provide trophic and possibly metabolic support for progenitor cells, and channel proteins, such as aquaporins, are thought to be important for determining water flux at the ventricle wall^11^. The speed of flow in the paravascular and in the glymphatic system has been reported; the average velocity of the fluid flow in the paravascular spaces of the mice brain is directly estimated as 17 μm/s^12^, and the fluid flow across the glial boundary of the brain tissue is estimated as 2 μm/s^13^.

The CSF production and turnover rates decline during senescence in human, which may result in impaired clearance of noxious substances (e.g., Aβ)^14^. The choroid plexus secretes CSF into the ventricles, and the choroid plexus (CP) has the potential to diminish Aβ levels and reduce Aβ-induced neurotoxicity; the transplantation of CP epithelial cells (CPECs) into an AD mouse brain revealed a significant reduction in Aβ deposits in the brain, which suggests that CPECs are involved in the Aβ clearance system^15^.

Cerebrospinal fluid-contacting (CSF-c) neurons are present in vertebrates, CSF-c expresses GABA and somatostatin. CSF-c neurons respond to both mechanical stimulation and to low pH^16^. The ventricular of the brain is filled by CSF, and the ventricle is lined with ependyma, whose apical surface is covered by motile cilia and CSF-c neurons. These suggest a possibility that certain class of neurons (e.g., CSF-c) are exposed and probably response to medium flow directly in the brain.

In the brain of a model mouse of AD (APPswe/PS1deltaE9), a normal sleep–wake circadian cycle in interstitial fluid (ISF) Aβ is seen before Aβ plaque formation. Following plaque formation, the sleep–wake cycle markedly deteriorated and circadian cycle dissipated, which is also seen in young adult humans with presenilin mutations^17,18^.

Until now, little has been known about the circadian rhythm in ciliary beating in mammals except the circadian rhythm of the ciliary beating frequency in human bronchial epithelial cells^19^. In this study, rat brain wall explant-cultures containing neurons and ependymal beating cilia were made. The neurons migrated from the explant were exposed to fluid flow (10 μm/s) induced by the beating of cilia. In vitro explant-culture of neurons and ependymal ciliary cells was used to examine the effect of medium flow on the neurotoxic action of Aβ1–42 in this study. We constructed a high speed imaging system to measure the frequency of the beating cilia, and observed the circadian rhythm of the beating frequency of ependymal cilia in the ventricle wall of newborn rats. Aβ1–42 at low concentrations affected the circadian rhythm of ciliary beating, and a neurotoxic effect of Aβ1–42 was diminished when neurons were exposed to fluid flow induced by the beating of cilia or by flow generated by a parallel-plate flow chamber system. These results suggest that Aβs decreases the peak frequency of ciliary beating, decreases medium flow, and enhances the Aβ-neurotoxicity by reducing the medium flow.

## Materials and methods

Briefly, Wistar rats (CLEA Japan, Inc., postnatal 4–10 days) were kept under a condition of 12 h light and 12 h dark. Whole brains were dissected out, and the ventricular walls of the brain ventricles, including the lateral, third, and fourth ventricles were dissected from 4 to 10-day-old rats, and cut into small fragments (approximately 500 µm cubic size) with scalpel blades after rapid decapitation under cold anesthesia^20^. The fragments of the brain were seeded on a 96-well culture plate (Primaria, Corning Inc., USA) with high glucose Dulbecco’s modified Eagle’s medium (DMEM), 10% fetal bovine serum (Equitech-Bio, Inc., USA) and penicillin (100 U/mL)-streptomycin (0.1 mg/mL) (Wako, Japan), and were kept in a 5% CO_2_ incubator (35 °C). The ciliary beating was observed on one side of the wall of the explant in the majority of the cases, and the neurons and glial cells were cultured for up to two weeks^21,22^. The beating frequency and amplitude were essentially the same throughout the observation period indicates that these cells were alive and beat cilia during the period (1–14 days), suggesting that simply culturing the explant does not significantly affect the health of the ependymal cells. Immunostaining of neurons and glial cells in the explant culture for 7 to 14 days and the live/dead assay of these cells showed that neurons and glial cells migrated from the explants and these cells were alive and healthy. Differential interference contrast images also confirmed that these cells were healthy. All the experimental protocols and animal handling were approved by the Animal Care and Use Committee of Kanazawa Institute of Technology, and follows the recommendations in the ARRIVE guidelines; thus, all methods were carried out in accordance with relevant guidelines and regulations.

### Measurement of the ciliary beating frequency

Reciprocal movement of the beating cilia in the explant culture was imaged by a differential interference contrast microscope (Nikon TMD-300) equipped with a temperature control unit and a cooled CCD camera (Orca-Flash 4.0, Hamamatsu Corporation, Japan); high-speed time-lapse imaging, 240 images (2048 × 256 pixels)/s, was performed. The explant cultures were stored under complete darkness in a conventional 5% CO_2_ incubator. The specimens were taken out of the incubator every four hours and time lapse imaging was performed with an inverted microscope for several minutes.

As shown in the supplementary movie 1, the beating motion of individual cilia was observed under a high-speed video microscope. The frequency of oscillation of individual cilia was almost identical, and the frequency of beating cilia was estimated by measuring the brightness of an area (10 µm × 10 µm) covering several tens of cilia. The fact that the beating frequency and amplitude were essentially the same throughout the observation period indicates that these cells were alive and beat cilia during the period (1–14 days). The beating frequency of the cilia (ciliary beating frequency, CBF)^23^ was measured and analyzed with software (HCimage 4.3.1.3, Hamamatsu Corporation, Japan, and ImageJ 1.53t ImageJ NIH, Bethesda, MD, USA); Links https://hcimage.com/ and https://imagej.nih.gov/ij/download.html. Imaging was performed at 35 °C.

In the separate experiment, brain wall explants with beating cilia were acutely isolated from newborn rats every 4 h and ciliary beating frequency (CBF) was measured; i.e., six animals were required for a 24-h CBF analysis.

### Reagents for experiments

Synthetic rat Aβ1–40 and rat Aβ1–42 (MedChemExpress Inc., USA) were diluted to the culture medium before use. In a typical experiment, Aβs were applied to 7-day-old brain explant culture and imaged daily for 7 days. Aβ1–42-conjugated tetramethylrhodamine (TAMRA) (AnaSpec, USA) was dissolved in ammonium hydroxide (1%) to a final concentration of 400 µM, and diluted into the culture medium at 0.4 µM before use with non-label Aβ1–42 at 3–10 µM. Forty-eight hours after the commencement of the treatment, the specimen was fixed in 4% paraformaldehyde for 10 min at room temperature, and TAMRA-Aβ1–42 was visualized with a Nikon fluorescence microscope (DIAPHOT-300 with 10× , 20× , and 40× objective lens, Nikon). For the quantitative analysis of the neurotoxicity of individual neurons migrated from the brain explant, neurons were counted. Phosphoramidon (20 μM, Santa Cruz Biotechnology, Santa Cruz, CA, USA), a metalloprotease inhibitor was added to the culture medium containing Aβs to inhibit neprilysin enzymatic activity^15^, except where otherwise mentioned in the text.

### Staining of samples

For immunocytochemistry, cells were fixed with 4% paraformaldehyde, and incubated overnight at 4 °C with mouse anti-β-III-tubulin (1:100; Promega, USA) and with donkey anti-mouse IgG 488 (1:100; FluoProbes, USA), which showed the majority of large soma with neurites were neurons. The shrunken (damaged) neurons were stained with trypan blue 0.04% (30 min treatment) and live/dead staining kit (Thermo Fisher scientific, USA), which stained the dead neurons in red. The live cells (green) and dead cells (red) were observed with a confocal microscope (Olympus, Fluoview). The dead neurons were counted manually^24^. We also used the following primary antibodies: anti-GFAP rabbit IgG (1:250, GeneTex GTX108711, USA) and anti-MAP2 chicken IgY (1:2000, abcam, MA, USA, ab5392). Secondary antibodies were Alexa Fluor 488-conjugated donkey anti-rabbit IgG (1:300, abcam, MA, USA, ab175674), and Alexa Fluor 405-conjugated goat anti-chicken IgY (1:300, abcam, MA, USA, ab150077).

### Devices for controlled shear stress

A concentric parallel-plate rheometers-based flow chamber system (parallel-plate flow chamber system) was employed to apply controlled shear stress to the brain explant culture; a glass rod (4 mm ϕ) was rotated 10 rpm 1 mm above the bottom of the 96-well culture plate^25^. Explant cultures were placed 1.5 mm away from the center of the rotating axis, which applied nearly uniform shear stress over the explant tissue and neurons in its vicinity as illustrated in Fig. 4B.

The flow of the medium 10–200 μm above the culture plate was directly measured by imaging the 2 μm polystyrene beads (Polyscience, USA) suspended in the flowing medium, which confirmed the magnitude of the shear stress; e.g., 15 μm/s at 10 μm above the culture plate in the vicinity of the beating cilia, corresponding to 1 mPa. Nearly the same shear stress was located to neurons in the vicinity of the beating cilia. This shear stress is less than 1/1000 of the stress that induces fluid shear stress injury^26^. In the parallel-plate flow chamber experiment, sham controls underwent the same protocol except for the rotation of the rod. In some experiment the experimental setup was modified as shown in Fig. 4B to apply 1 mPa shear stress only to Aβ1–42-containing medium; a 4-mm square coverslip was placed 1 mm above the explant culture, which prevents the transfer of the shear stress to neurons. Aβ1–42 was applied to the neurons placed behind the coverslip by diffusion and very slow medium flow (0.4 μm/s).

A stage heater (PT-100, Nikon, Japan) maintained a constant temperature (35 °C) and images were taken with the inverted microscope. The experiments were performed more than three times unless otherwise noted. Student’s t-test and Two-way (or one-way) ANOVA (Origin ver. 2020b, Origin software, USA) were used in the statistical analysis; Link, https://www.originlab.com/.

### Significance statement

Alzheimer’s disease (AD) is a progressive dementia accompanied by the accumulation of Aβ1–42 and a decreased clearance rate of Aβ1–42. Studying the action of Aβs on the Aβ clearance system is important to understand AD development. We report the circadian rhythm of the beating frequency of ependymal cilia. The beating frequency peaked at midday, and was decreased by Aβ1–42. Since the ciliary beating is involved in the clearance system of Aβ, the results suggest that the clearance system in the brain is impaired by Aβ1–42. In addition, the neurotoxicity of Aβ1–42 was reduced by cilia-mediated flow; Aβ1–42 accordingly enhances Aβ1–42 neurotoxicity by reducing the flow.

## Results

### The inhibitory action of Aβs on the circadian rhythm of the beating frequency of ependymal cilia

**Figure 1:**
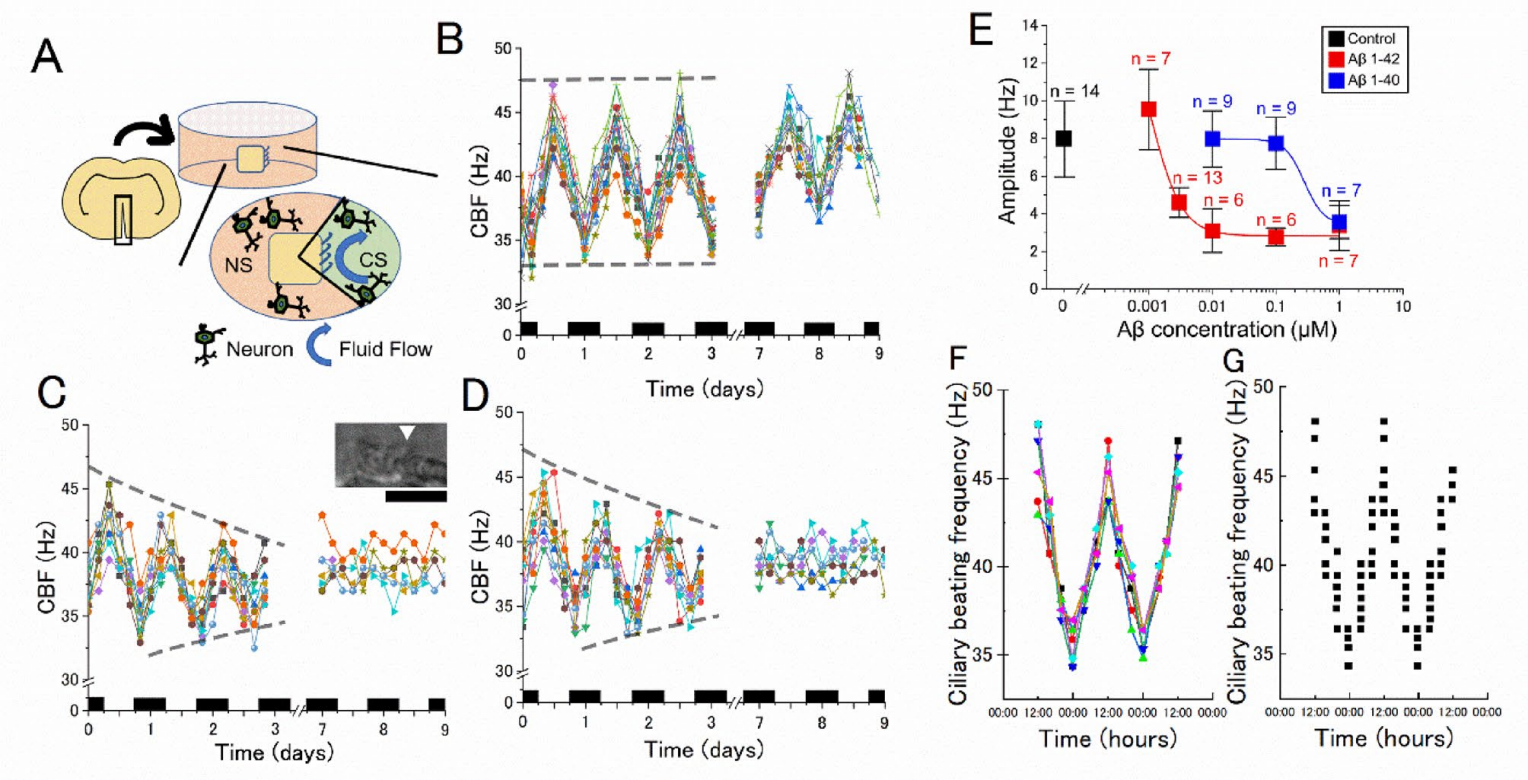
Inhibitory action of Aβs on the circadian rhythm of the ciliary beating frequency. (**A**) A schematic drawing of the brain explant culture from the brain wall. Neurons migrated from the brain explant are illustrated schematically. CS, ciliated side; NS, non-ciliated side. (**B**) The circadian oscillation of the frequency of the ciliary beating in the control medium. (**C**) The circadian oscillation in 10 nM Aβ1–42. (**D**) The circadian oscillation and in 1 µM Aβ1–40. The vertical axis is the ciliary beating frequency (CBF) and the horizontal axis is the time (days) from the start of imaging (2–3 days after making the explant culture). Black bars denote the night-time (6:00 p.m.–6:00 a.m.). The ependymal cilia were kept under a constant condition (35 °C, 5% CO_2_ concentration, in total darkness) and the frequency of beating was measured every 4 h with the optical microscope. Each color denotes an individual explant culture. The CBF of 16 explant cultures was recorded on day 0 and 14 cultures on day 7 in panel (**B**). The inset in panel C shows typical ciliary cells on the wall of a chemically fixed explant culture. Bar, 10 µm. (**E**) The concentration dependent inhibitory action of Aβs on the amplitude of the circadian rhythm. The amplitude of the circadian rhythm (i.e., the difference between the maximum and the minimum frequency of the ciliary beating) was measured on day 7 in the control medium and under different concentrations of Aβ1–42 (red) and Aβ1–40 (blue). Error bars denote the standard deviation of the mean (the number of data points shown near the bar). (**F**) The CBF recorded from these pieces of acutely dissected brain tissue showed a circadian rhythm with a 24-h period. Each color corresponds to the CBF of a piece of dissected brain tissue with ependymal cilia prepared at 12:00. (**G**)The distribution of CBF recorded from acutely dissected brain tissue with ependymal cilia when the dissection was made every four hours. Each point corresponds to a piece of dissected brain tissue. Timepoints of the first 12:00–12:00 (24 h) have been duplicated to facilitate viewing of the time curve. N denotes the number of culture wells. Image analysis by HCimage and ImageJ.

The beating frequency of the ependymal cilia of rat explant brain tissue cultures of the ventricular walls was measured over four consecutive days (see more materials and methods), which revealed a cyclic pattern; the frequency peaked at noon each day (48 ± 1.9 Hz, n = 16 explant cultures) and decreased at midnight (36 ± 1.6 Hz, n = 16). Fourier analysis showed a period of 25.5 h (n = 16), indicating the presence of a circadian rhythm of the ciliary beating frequency under the explant culture condition (Fig. 1). This circadian rhythm was stably observed for 14 days. The fact that the beating frequency and amplitude were essentially the same throughout the observation period indicates that these cells were alive and beat cilia during the period (1–14 days), suggesting that simply culturing the explant does not significantly affect the health of the ependymal cells.

Furthermore, brain wall explants with beating cilia were acutely isolated from new born rats every 4 h and ciliary beating frequency (CBF) was measured, which showed the circadian rhythm with a cycle length of 24 h (Fig. 1G) (data collected from three independent experiments). After 2 days of storage in explant culture, the circadian rhythm of CBF peaked at noon (Fig. 1F), suggesting the existence of a circadian rhythm not only in vitro but also in vivo.

In the presence of Aβ1–42, the circadian rhythm of CBF was obscured within 9 days, as the maximum CBF gradually decreased and the minimum CBF slightly increased. There was a difference of 12 Hz (from 36 to 48 Hz), named the amplitude of the rhythm, in the control condition, but it decreased to a few Hz in Aβ1–42. The amplitude of the circadian rhythm of CBF decreased in a dose-dependent manner and was halved at about 3 nM (Fig. 1). Aβ1–40 also decreased the amplitude of the circadian rhythm, as shown in Fig. 1, and was halved at 0.3 μM, showing that Aβ1–42 is two orders of magnitude more potent. It should be noted that both Aβ1–42 and Aβ1–40 did not stop the beating itself; 40 Hz ciliary beating with almost the same amplitude was observed. The cycle length of the circadian rhythm (ca. 24 h) was apparently unaffected by Aβ1–42 (1 nM to 1 μM) and Aβ1–40 (10 nM to 1 μM).

### Neurotoxicity of Aβ1–42 was detected in neurons of the brain explant culture, but was less evident in neurons in the vicinity of beating cilia

**Figure 2:**
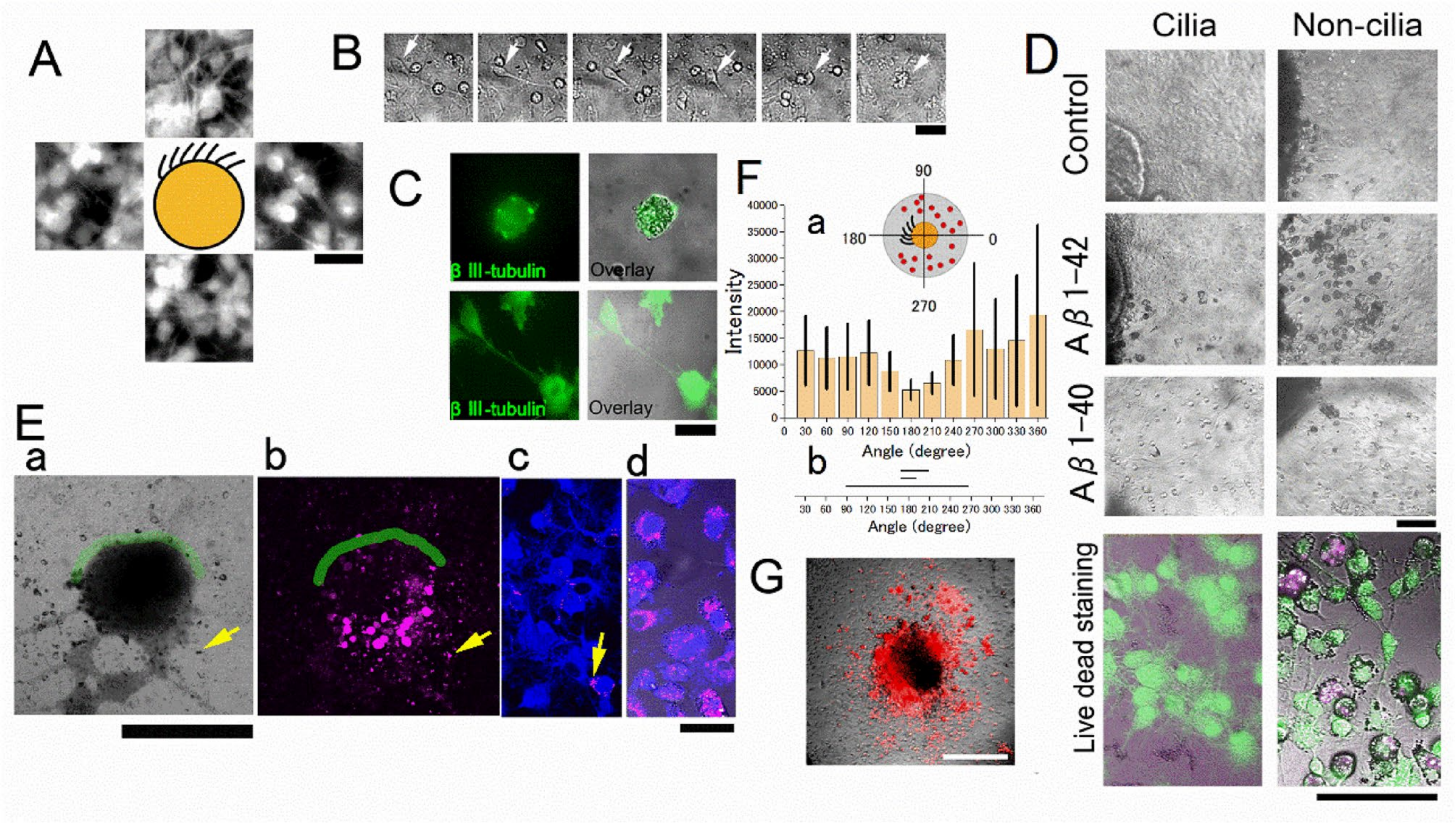
Neurotoxic effects of Aβ1–42 observed in the explant culture. (**A**) Fluorescence images of tubulin βIII positive neurons migrated from the explant; images are from the area 0.5 mm up and down, left and right of the schematically illustrated explant. (**B**) Typical time-lapse images of neurites retracted in 10 µM Aβ1–42 (time-lapse images were taken at 1, 2, 3, 4, 5, and 13 h from left to right). The soma is shown by the arrows. (**C**) A typical shrunken neuron in 3 µM Aβ1–42 for three days is positive for tubulin βIII (left) and superimposed on the DIC image (right). Neurons in the control medium in the same notation (lower panels). (**D**) TB-positive shrunken cells in the explant culture in the Aβs. DIC images of explant culture of the control (upper), Aβ1–42 (3 µM, middle), and Aβ1–40 (3 µM, lower). Live (green) and dead (red) cell staining of neurons superimposed on the DIC images (10 µM Aβ1–42 for four days, bottom) on the cilia side (left column) and non-cilia side (right column). (**E**) A typical explant culture treated with Aβ1–42 (10 µM) and RAβ1–42 (0.4 µM). (a) DIC image of an explant culture. The yellow arrow shows a typical shrunken cell. (b) Fluorescence image of RAβ1–42. The shrunken cell positive for RAβ1–42 is pointed by the arrow. (c) Neurons on the ciliated side are positive for MAP-2 (blue). Small fraction of neurons positive for RAβ1–42 (magenta) shown by an arrow. (d) Neurons on the non-ciliated side are positive for MAP-2 and RAβ1–42. The green line shows the ependymal cilia cells (a and b). (**F**) (a)The distribution of RAβ1–42 plotted in the polar coordinate system of the explant culture; the fluorescence intensity of RAβ1–42 in the area 100 μm from the edge of the explant culture was plotted. N = 3 culture wells. The inset is an illustration of an explant culture and RAβ1–42 positive cells (red dots) and the assignment of the angle; the polar coordinate system of the explant culture is divided into 12 sections [ 0, 30), [ 30, 60) …, [ 330, 360); the center of the ciliated area is assigned 180 degrees. (b) The individual bar denotes the distribution of beating cilia. The fluorescence intensity of RAβ1–42 on the non-ciliated area is significantly higher than that on the ciliated area control (*p* = 0.006, one-way ANOVA test, Origin ver. 2020b). (**G**) fluorescence image of RAβ1–42 positive cells of an explant culture that had no ciliated cells. Bars are 50 µm (panel a) and 30 µm (panels **C**), 100 µm (panels **B** and **D**), 500 µm (panels **E** a and b), 50 µm (panels **E** c and d), and 300 µm (panel **G**). N denotes the number of culture wells.

Neurons with neurites migrated from the brain explant and were distributed uniformly on the bottom surface of the culture wells as shown in Fig. 2A. The average density of migrated neurons on the ciliated side of the culture within 100 μm from the surface of the explant culture was 885 ± 267 cells/mm^2^ (n = 5) and on the non-ciliated side was 1011 ± 275 cells/mm^2^ (n = 5), with no significant difference between the two sides (schematically illustrated in Fig. 1A). The number of migrated cells gradually decreased with the distance from the surface of the explant. The results of immunostaining and live/dead assays on the brain explant after 7 to 14 days of culture showed that neurons and glial cells were alive and had migrated from the explant. The reduced height of the explant during this time, likely due to the migration of these cells, indicates that they were in good health.

In the presence of Aβ1–42 (3–25 µM for 7 days), the number of round-shaped neurons increased, especially on the non-ciliated side of the explant culture. The mean percent of round-shaped neurons on the ciliated side of the culture within 100 μm from the surface of the explant culture was 8.03 ± 5.93% (n = 4), which was significantly (*p* = 0.02, t-test) less than 20.0 ± 3.70% (n = 4) on the non-ciliated side in the presence of 10 μM Aβ1–42. These values were 4.10 ± 3.50% (n = 5) and 6.47 ± 3.48% (n = 5), respectively, in the control experiment without Aβ1–42. Time-lapse imaging of the neurons showed the neurites were retracted in Aβ1–42 (Fig. 2B). The round-shaped neurons were stained with trypan blue (TB) and stained “red” with live/dead staining kit, and many granules were found in the cell body in the differential interference contrast (DIC) images (Fig. 2D). The concentration of Aβ1–42 (10 µM) was 2 orders higher than the concentration (0.03 µM) that affected the circadian rhythm of ciliary beating frequency. On the other hand, Aβ 1–40 (1 to 10 µM) had no significant toxic effect on the neurons.

Round-shaped neurons with shrunken soma (named shrunken cells) on the non-ciliated side of the explant culture (Fig. 2C) were stained with rhodamine-labeled Aβ1–42 (RAβ1–42) (Fig. 2E). The RAβ1–42-positive neurons were distributed on the non-ciliated side (Fig. 2Ed), and the chance to find RAβ1–42-positive neurons was diminished on the ciliated side (Fig. 2Eb and F). In addition, the RAβ1–42 inside the explant brain tissue showed neurons near the beating cilia were less stained than neurons in the other parts of the explant tissue (Fig. 2Eb). This suggests that Aβ1–42 is taken up by the neurons on the non-ciliated side, while Aβ1–42 is less efficiently taken up by neurons on the ciliated side (Fig. 2Ec and d). When the brain explant cultures of the non-ciliated part of the brain near the ventricle wall were treated with RAβ1–42, RAβ1–42-positive shrunken cells were uniformly distributed inside and outside the explants (Fig. 2G).

### Analysis of the medium flow by cilia and its effect on the neurotoxic action of Aβ1–42

**Figure 3:**
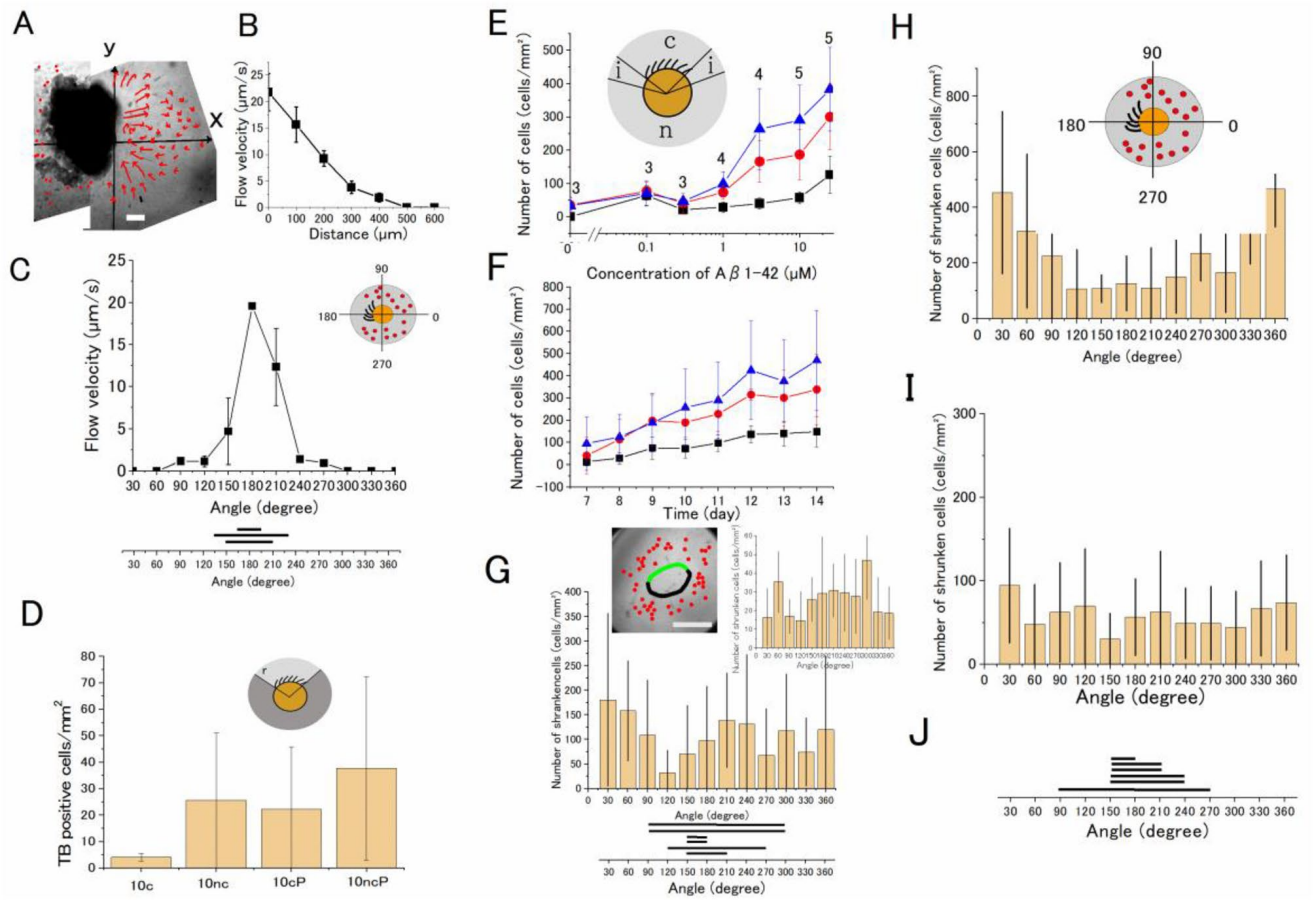
Analysis of the toxic effect of Aβ1–42 diminished on the ciliated side of the explant culture. (**A**) A flow map of the medium around a brain explant with beating cilia. The flow directions are indicated by arrows. The length of the arrow denotes the speed of flow estimated by particle tracking at 110 µm above the substrate for 1 s. (**B**) The speed of the flow along the x-axis at 10 µm above the substrate; the speed is high near the beating cilia (x = 0) and declines with distance from the explant. N = 3. Bars denote the standard deviation of the mean. (**C**) The distribution of the speed of the flow at 10 µm above the substrate plotted in the polar coordinate system of the explant culture (inset). N = 3. Bars denote the standard deviation of the mean. (**D**) The neurotoxic effect of Aβ1–42 was augmented by phosphoramidon (20 µM). The number of TB-positive cells in mm^2^ in 10 µM Aβ1–42 on the ciliated side of the explant culture (10c), in 10 µM Aβ1–42 on the non-ciliated side of the explant culture (10nc), the number of TB-positive cells in 10 µM Aβ1–42 with 20 µM phosphoramidon on the ciliated side (10cP), and that on the non-ciliated side (10ncP). The number of TB-positive cells in 1 mm^2^ on the non-ciliated side is higher than that on the ciliated side, and that in phosphoramidon is significantly higher than that in the control (*p* = 0.04, two-way ANOVA test). (**E**) The dose-dependent increase in the neurotoxicity of Aβ1–42 on the non-ciliated (blue triangle), ciliated side (black squares), and the intermediate area (red circles) between the ciliated and non-ciliated sides of the explant culture. These regions are shown in the inset; “c” cilia, “i” intermediate (20 degrees in the polar coordinate system), and “n” non-cilia regions. The number of data points is shown in the figure. The number of shrunken cells in 1 mm^2^ on the ciliated, non-ciliated, and intermediate area was significantly different (p = 9.6 × 10^−7^, two-way ANOVA test), supporting the idea that the flow affects the Aβ1–42 neurotoxic action. (**F**) Time-dependent increase in the neurotoxicity of Aβ1–42 on the non-ciliated (blue triangle), ciliated side (black squares), and the intermediate (red circles) region. The number of data points is 5 (except 3 on day 12). The interaction between the time-dependent increase in the toxic effect of Aβ1–42 and the flow level was not significant, suggesting that the time delay of the Aβ1–42 neurotoxic action was not apparently affected by the medium flow. (**G**) When the explant brain tissue was removed from the bottom and 25 µM Aβ1–42 was applied, a nearly uniform distribution of shrunken cells was seen in the polar coordinate system of the explant culture. Inset image, the circle denotes the position of the pre-existing explant, and the green line denotes the distribution of pre-existing beating cilia. Bar denotes 500 μm. The number of shrunken cells within the area 100 μm from the edge of the explant culture was counted. N = 6. Inset graph, the number of shrunken cells increased from 7 to 14 days culture within 350 μm from the edge. The horizontal bars at the bottom denote the distribution of beating cilia in the polar coordinate system. These distributions of the number of shrunken cells are not dependent on the pre-existing cilia of the removed explant. (**H**) The distribution of shrunken cells in the polar coordinate system of the explant culture in 25 µM Aβ1–42. The number of shrunken cells in the area 100 μm from the edge of the explant culture was counted. The inset shows the polar coordinate system and the assignment of the angle. (**I**) The distribution of shrunken cells in the area 100–350 μm from the edge of the same set of explant cultures in (H). N = 6. (**J**) The horizontal bars denote the distribution of beating cilia in the polar coordinate system. The number of shrunken cells in the ciliated area is lower than that of the non-ciliated direction in panel (**H**) (p = 5.8 × 10^−5^, two-way ANOVA test). N denotes the number of culture wells. Statistical analyses by Origin ver. 2020b.

On the ciliated side of the explant culture, the speed of the flow at 10 µm above the migrated neurons was 22 µm/s in the vicinity of the beating cilia (Fig. 3A), which is in the same order of magnitude as reported recently^27^. The flow was greatly diminished at 300 µm away from the beating cilia (Fig. 3A) and was not detected on the non-ciliated side (Fig. 3B, C). The neurotoxic effects of Aβ1–42 were observed in neurons on the non-ciliated side and were reduced in neurons on the ciliated side when migrated neurons within 100 µm from the explant tissue exposed to flow (> 15 µm/s) were examined (Fig. 3H). On the other hand, the neurotoxicity of Aβ1–42 was almost uniformly observed (Fig. 3I) in neurons 100–350 µm away from the explanted tissue exposed to a lower magnitude of flow. The distribution of beating cilia is shown in Fig. 3J.

When explant tissue with beating cilia was removed from the culture plate and Aβ1–42 was applied, Aβ1–42 neurotoxicity was uniformly detected (Fig. 3G). This indicates that neurons migrated from regions of the explanted brain tissue are sensitive to Aβ1–42 irrespective of the original position of neurons before migration.

The dose-dependent effect of Aβ1–42 was detected in neurons in areas with or without cilia and the intermediate area (see illustration in Fig. 3E). The dose-dependent toxic effect of Aβ1–42 was detected with nearly the same half-maximum concentration (Fig. 3E). The time-dependent effect of Aβ1–42 was examined in the same way; the time dependent development of the toxic effect was nearly the same in all three areas (Fig. 3F), suggesting that the time dependent process of the Aβ1–42 neurotoxic action was not apparently affected by the medium flow.

The neurotoxic effect of Aβ1–42 was augmented by phosphoramidon (20 µM), a neprilysin inhibitor, on both the ciliated and non-ciliated side of the explant culture (Fig. 3D), supporting that oligomerized Aβ1–42 was toxic in the explant cuture as has been reported^28^.

### Effects of the artificial flow on the Aβ1–42 neurotoxicity

**Figure 4:**
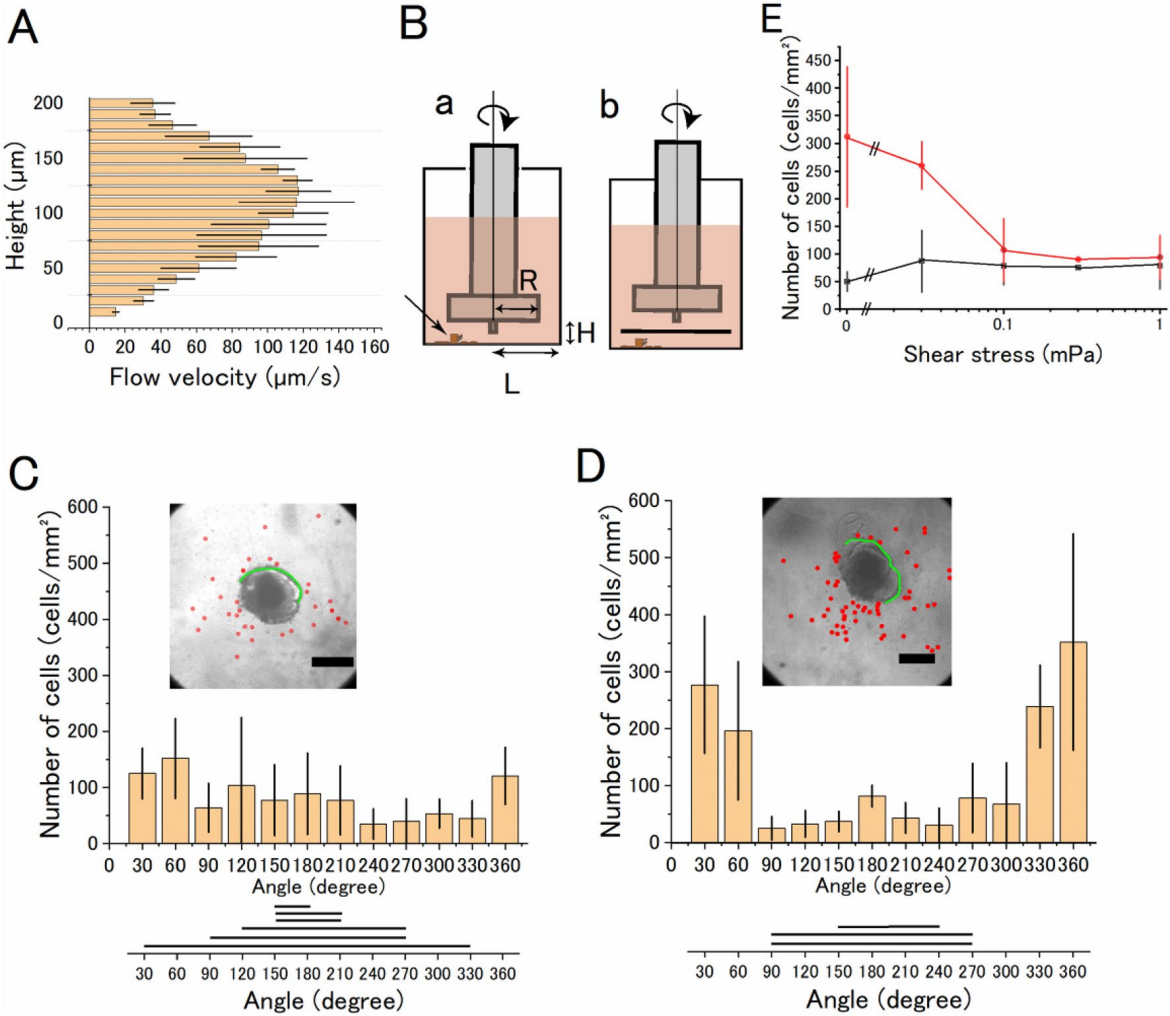
Effects of artificially generated medium flow to neurons migrated from the explant brain tissue culture with Aβ1–42. (**A**) The flow map of the medium along the z-axis of the explant brain tissue culture with beating cilia shows that the speed of flow in the vicinity (ca. 10 μm) of the beating cilia is high at around 100 μm from the bottom where the beating of cilia was detected and is low at the bottom of the culture plate. N = 3. (**B**)(a) A schematic drawing of the parallel plate flow chamber and an explant culture. H = 1 mm, R = 2 mm, and L = 3 mm. The arrow shows the position of the brain explant. (b) A 4-mm square coverslip was placed 1 mm above the neurons. (**C**) The distribution of shrunken cells in 10 µM Aβ1–42 under 1 mPa shear stress in the area 100 μm from the edge of the explant culture in the polar coordinate system. The inset in panel (**C**) shows a typical DIC image of the explant culture in 10 µM Aβ1–42 under shear stress for four days. The inset in (**D**) shows the explant culture of the sham control. Red dots denote the location of the shrunken cells. The green line denotes the area of ciliary cells found. Bar, 500 μm. The distribution of shrunken cells on the ciliated or on the non-ciliated side was significantly different. The distribution of shrunken cells in the presence or absence of the artificial flow was also significantly different (*p* = 0.04, two-way ANOVA test, Origin ver. 2020b). (**E**) Shear force dependent decrease in the neurotoxic action of Aβ1–42 (10 µM). Vertical axis, number of dead cells/mm^2^ on the non-ciliated side (red circles) and ciliated-side (black circles), and horizontal axis, shear stress. N = 3, except N ﻿ = 1 at 0.3 mPa (N﻿ denotes the number of culture wells).

The profile of the flow speed of the medium generated by beating cilia was measured at heights of 10–200 μm above the migrated neurons (at a distance of 10 μm from the beating cilia) in order to estimate the shear stress to the neuron on the glass surface. The flow speed increased with increasing distance from the bottom in the range of 0–100 μm and decreased (100–200 μm) as shown in Fig. 4A. The flow speed was 15 μm/s at 10 μm above the bottom of the culture dish, which corresponds to a shear stress of 1 mPa (drag force acts on the cell surface). Shear stress (0–1 mPa) was artificially generated by the parallel-plate flow chamber system to mimic the flow by cilia (Fig. 4B), and the effect of shear stress on neurons was examined.

When the explant culture neurons were subjected to a shear stress of 1 mPa for 4 days, the shape of the explant culture, beating frequency of the cilia, and the distribution of neurons migrated from the brain explant were not affected. In the presence of Aβ1–42, the density of the shrunken cells on the non-ciliated side was decreased (Fig. 4C) compared with the sham controls that underwent the same protocol except for rotating the rod (Fig. 4D). The neurotoxic effect of agitated Aβ1–42-containing medium used to apply shear stress was observed after 4 days of treatment, indicating that Aβ1–42 retained its neurotoxicity even under shear stress, but neurotoxic action on neurons was weakened by the artificial flow. A dose-dependent reduction in the neurotoxic effects of Aβ1–42 was seen at shear stresses ranging from 0.03 to 0.3 mPa, with the effect saturating at shear stress above 0.3 mPa (n = 3) (Fig. 4E).

The conformation of Aβ1–42 might be changed by flow^29^, which may affect the Aβ-neurotoxicity for short period of time (e.g., minutes to hours). The experimental setup was modified as shown in Fig. 4Bb to apply 1 mPa of shear stress not to the neurons but to the Aβ1–42-containing medium. In this setup, Aβ1–42 spread diffusively over the neurons placed behind the coverslip within 5 min. The neurotoxic effect of Aβ1–42 was observed (Supplemental Fig. 1S) in a similar way to the sham control, supporting the idea again that the agitated Aβ1–42 retained its neurotoxic action.

## Discussion

The circadian rhythm of the ciliary beating frequency (CBF) of rat ependymal cilia of an explant brain tissue culture was detected. The beating frequency was high during the period of sleep (midday) and was low during the period of being awake (midnight), if rats were assumed to be alive. Aβ1–42 reduced the peak frequency of the beating at midday, resulting in the circadian rhythm becoming obscure. Aβ1–40 also reduced the amplitude of the circadian rhythm but with less potency. Aβ1–42 exhibited neurotoxicity to neurons on the non-ciliated side of the explant culture, while the neurotoxicity was less evident on neurons on the ciliated side. This observation was reinforced by the fact that neurons on the non-ciliated side were stained by rhodamine-labeled Aβ1–42 but neurons on the ciliated side were less stained. The neurotoxic effect of Aβ1–42 on neurons on the non-ciliated side was diminished when neurons were exposed to shear stress of 1 mPa generated artificially by the parallel-plate flow chamber system, which mimics the medium flow by cilia. These results demonstrate that Aβ1–42 affects the circadian rhythm of the ciliary beating of the ependymal cilia, and decreases the beating frequency of the cilia approximately 10% during sleeping period, which decreases the fluid flow. These observations suggest the possible enhancement of the Aβ1–42 neurotoxicity by Aβ1–42 itself in the brain explant culture by reducing the medium flow. These Aβ effects may play a role in the brain in vivo as discussed below.

Brain-derived Aβ can be transported into the peripheral pool via the brain blood barrier (BBB), blood–CSF barrier, arachnoid villi, or the glymphatic–lymphatic pathway^3^. The Aβ concentration in the brain is ca. 10^3^ times higher than CSF^3,7,30^. When a certain fraction of Aβ is transported to the peripheral pool via CSF, brain-derived Aβ is diluted in CSF and transported/diffused to the CSF absorption clearance system (e.g., via arachnoid villi, the blood-CSF barrier, or the glymphatic–lymphatic pathway). Therefore, at least the following three factors are important for Aβ clearance from the brain (1) the total volume of CSF (or production of CSF by choroid plexus) for Aβ dilution, (2) mixing of CSF by ciliary beating for Aβ diffusion/dilution, and (iii) the CSF absorption/clearance system. Ideas (1) and (2) are supported by the observation that the transplantation of choroid plexus epithelial cells into an AD model mouse brain, which revealed a significant reduction in brain Aβ deposits^15^. The motile cilia beating generates localized CSF flow. It has been proposed that local CSF flow generated by highly motile cilia clears debris from the ventricle walls as well as enhances mixing^31^.

In live mice, natural sleep is associated with a 60% increase in the interstitial space, resulting in a marked increase in convective exchange between CSF and ISF, which increased the rate of Aβ clearance during sleep^32^. This presumably causes the amount of Aβ levels in the ISF to be high while awake and low while asleep^18^. Our observations of the circadian rhythm in the CBF of rat ependymal cilia is consistent with the above idea that the high-frequency beating during the sleep period of the rat (midday) is associated with an increase in the rate of Aβ clearance. The low-frequency beating during the awake period (midnight) is associated with a decrease in the rate of clearance, causing the accumulation of Aβ1–42 and reduction in the CSF medium flow. These changes will lead to a decrease in the ISF/CSF circulatory system, which may enhance Aβ1–42 toxicity. These ideas are supported by the fact that the stasis of flow is observed in the brain of the animal model of hydrocephalus, and deposits of Aβ1–42 is facilitated^33^.

Increased Aβs in CSF during the very early phase of cerebral Aβ deposition in mouse AD models has been recently reported^34^. The Aβ1–42 concentration in APP24 AD model mice increases from 10,000 to 13,000 pg/mL^34^, which corresponds to an increase from 2.2 to 2.8 nM. This increase in the Aβ1–42 concentration may affect the circadian rhythm of the ciliary beating, according to our observations, and we consider a new possibility that the increase in Aβ1–42 in the early phase of AD reduces the peak frequency of the cilia beating and decreases the rate of Aβ clearance during sleep, and leads to accumulation of Aβ1–42 in the brain as well as it enhances the Aβ neurotoxicity. If the circadian rhythm in CSF flow affects the awake and sleep pattern, then the inhibitory effect of Aβ1–42 on the circadian rhythm in the ciliary beating may also be involved in the sleep pattern disturbance in AD model animals^35^ and in AD patients^36^. With the technological development of non-invasive recording of ependymal cilia beating^37^ in vivo, where a very small magnetic particle is attached to the beating cilia and the movement of the particle is detected with a very sensitive magnetic sensor, SQUID magnetic gradiometer placed above the animal's head. With this system and injection of Aβ into the brain ventricles, we will be able to study the relationship between the disruption of the circadian rhythm in ependymal cilia beating by Aβ, sleep disruption, and Aβ accumulation in the brain parenchyma under in vivo conditions. At present the precise cellular and molecular mechanism of the circadian rhythm of CBF has not been elucidated. Our specimen did not contain the suprachiasmatic nucleus, the circadian center of the brain. Thus, the rhythm may be driven by astrocytes^38^ or by ciliated ependymal cells themselves.

Aβ1–42 is neurotoxic, but the neurotoxicity was reduced on the ciliated side, where neurons were exposed to flow generated by the beating of cilia or by the parallel-plate flow chamber system. Aβ1–42 retained its neurotoxic effect under shear stress under our experimental conditions. These results suggest that neurons exposed to the flow become resistant to the neurotoxicity of Aβ1–42. The diminished neurotoxic action of Aβ1–42 was detected under the artificial flow causing shear stress more than 0.1 mPa (1.5 μm/s at 10 μm above the substrate). The average velocity of the fluid flow in the paravascular spaces of the mice brain is directly estimated at 17 μm/s^12^, and the fluid flow across the glial boundary of the brain tissue is estimated at 2 μm/s^13^. Assuming the flow in the brain tissue is 2 μm/s at 10 μm above the substrate (the steepest part of the dose–response curve), slight flow decline (ca. 10%) by Aβs possibly enhances the neurotoxic action of Aβs. A recent investigation of the fine structure of live brain extracellular space shows 80–270 nm between cells^39^, and the flow rate (2–200 nm/s)^27^ through the narrow space is estimated^40^, which suggest that neurons exposed to fluid shear stress in the 0.04 to 4 mPa range, assuming the radius of the cylindrical path, 140 nm. Thus, the shear stress (0.1 to 1 mPa) examined in this study may affect the neurotoxic action of Aβ1–42 in the in vivo brain. Neurons are very sensitive for shear stress compared with well-studied endothelial cells, since large chronic shear stress (sub Pa) affects the endothelial cells^41,42^.

The concentration in the medium is considered to be relatively stable and uniform due to the following reasons; oligomers of Aβ1–42 have large diffusion constants due to their small size (roughly in the range of a few nm, size of low-molecular-weight proteins)^43^; neurons are uniformly distributed, and Aβ1–42 was taken up almost exclusively by neurons; in addition, no significant adsorption of Aβ to the glass surface (cell substrate) was observed in this study. Neurons are uniformly distributed, and Aβ1–42 was accumulated almost exclusively in neurons with/without media flow. We did not find that certain types of neurons were selectively damaged by Aβ1–42 under these experimental conditions (Fig. 2D and E). The distribution of rhodamine-Aβ1–42 on the coverslip was examined. Two μL of culture medium from the explant cultured for four days with 0.4 μM rhodamine-labeled Aβ1–42 and 10 μM unlabeled Aβ1–42 was added to a small observation chamber. Aβ1–42 bound to the coverslip and fluorescent spots corresponding to monomer and oligomer of rhodamine-labeled Aβ1–42 were detected. The fluorescent spots of Aβ1–42 were randomly distributed and covered the surface of the coverslip (Supplemental Fig. 2S), supporting the idea that Aβ1–42 was uniformly distributed in the culture medium, thus Aβ1–42 was probably homogeneously distributed in the culture medium, and bound to the neurons at the bottom of the culture dish. Therefore, it is not likely that the uneven distribution of Aβ in the culture medium could cause high neuronal cytotoxicity on the non-ciliated side by increasing the local concentration of Aβ1–42. The oligomers of Aβ1–42 (especially small ones) mentioned above may interact with a high-affinity receptor for Aβ1–42 as reported in the preceding study^44^ and may have neurotoxic effects.

The results in Fig. 4 suggest that flow itself does not interfere with the neurotoxicity of Aβ. Flow rather increases the chance of the binding agonist and receptors in a certain cases^45^. Thus, these observations regarding flow cannot account for the decrease in the accumulation of Aβ in neurons on the ciliated side.

The cellular and molecular mechanism behind this inhibitory action of medium flow are not known at present. However, several possible effects of gentle flow on neurons are known. When cells were cultured under continuous flow, the neurite outgrowth during the differentiation of neural progenitor cells is augmented^46^. The continuous media flow may facilitate the supply of oxygen and nutrients, thereby protecting neurons from the neurotoxicity of Aβ1–42.

Amyloid-β-induced neuronal hyperexcitability is reported^47,48^. The neurons migrated from explant brain tissue may include CSF-c neurons which express GABA and somatostatin^16^. The fluid flow elicits action potentials in these neurons. Combining these studies suggest a possibility that ciliary beating elicit fluid flow, activate CSF-c neurons, diminish the Aβ1–42 induced hyperexcitation in neurons which receive GABAergic inhibitory input from the CSF-c neurons. It may be worth pointing out other possible mechanisms, which include: (1) the association of exosomes with Aβ^49^, crucial for Aβ-neurotoxicity, might be inhibited by flow; (2) flow may modify the Aβ1–42 (or the Aβ-exosome) binding to neurons, uptake at the cell surface, and diminish the Aβ1–42 neurotoxicity. These possibilities should be explored in future studies.

In this study, cilia maintained their rhythm for as long as 14 days in vitro. ATP-dependent sliding of microtubules and beating is demonstrated in the Triton-extracted axoneme^50^, and ATP-dependent ciliary beating was also observed in Triton-treated rat ependymal cells in long-term culture. These suggest that the ciliary beating mechanism functions stably under long-term culture conditions and is dependent on ATP-dependent sliding, and generally ATP levels are stably regulated in cells. These may explain why cilia maintain their rhythm for days in vitro. Little is known about the regulation of ciliary beating by endogenous biomolecules, e.g., neurotransmitters and peptides^51^. The present study adds new knowledge to the limited scientific literature on the regulation of ciliary beating.

### Supplementary Information

Supplementary Video 1.

Supplementary Figures.

## Data Availability

The datasets used and/or analysed during the current study available from the corresponding author on reasonable request.
