# Supplementary material for: Amyloid-β slows cilia movement along the ventricle, impairs fluid flow, and exacerbates its neurotoxicity in explant culture
Source: Sci Rep. 2023 Aug 21;13:13586. doi: 10.1038/s41598-023-40742-0 (PMC10442439; doi:10.1038/s41598-023-40742-0)
Supplement: Supplementary file 2 — Supplementary Figures. [file 41598_2023_40742_MOESM2_ESM.docx]

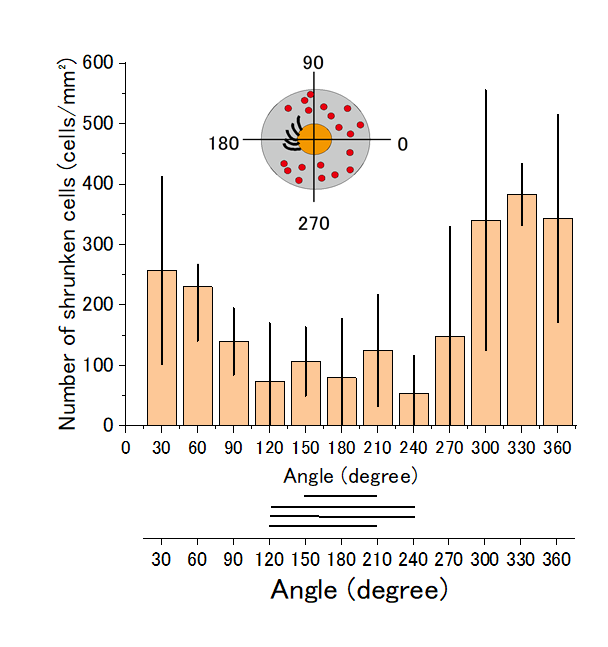


Fig. S1. Supplemental Figure 1. Aβ1-42 agitated by 1 mPa shear stress retained its neurotoxic action. Aβ1-42 was diffused to the neighboring neurons placed behind the coverslip slowly within 5 min as shown in Fig. 4Bb. The distribution of shrunken cells in 10 µM Aβ1-42 under shear stress in the area 100 μm from the edge of the explant culture in the polar coordinate system. The distribution of shrunken cells on the ciliated or on the non-ciliated side was significantly different (p=0.01, two-way ANOVA test).





Supplementary Figure S2. A fluorescence image of single R-Aβ1-42 molecules attached to the glass surface. The concentration of R-Aβ1-42 was 400 nM. A typical fluorescence image is shown by the yellow arrow (approximately thirty R-Aβ1-42 molecules are detected). Images were obtained with total internal reflection microscopy ^1^. Bar is 10 μm.

Supplemental movie 1. Time lapse imaging of beating cilia on the brain wall explant culture. Time-lapse images, 240 images/s total length 300 ms. The width of the image 50 μm.

1 Okura, K. & Tatsumi, H. Surface-dependent quenching of Qdot emission can be a new tool for high resolution measurements. *Scientific reports* **13**, 1869, doi:10.1038/s41598-023-28910-8 (2023).
